# Dimension-reconfigurable bubble film nanochannel for wetting based sensing

**DOI:** 10.1038/s41467-020-14580-x

**Published:** 2020-02-10

**Authors:** Yu Ma, Miao Sun, Xuexin Duan, Albert van den Berg, Jan C. T. Eijkel, Yanbo Xie

**Affiliations:** 1 International Joint Laboratory of Nanofluidics and Interfaces, School of Physical Science and Technology, Northwestern Polytechnical University, 710100 Xi’an, China; 2 MOE Key Laboratory of Material Physics and Chemistry under Extraordinary Conditions, School of Physical Science and Technology, Northwestern Polytechnical University, 710072 Xi’an, China; 3 State Key Laboratory of Precision Measuring Technology and Instruments, College of Precision Instrument and Opto-Electronics Engineering, Tianjin University, 300072 Tianjin, China; 4 BIOS Lab-on-a-Chip Group, MESA+ Institute for Nanotechnology, Technical Medical Centre and Max Planck Center for Complex Fluid Dynamics, University of Twente, 7522NB Enschede, The Netherlands

**Keywords:** Biomedical engineering, Nanofluidics, Fluid dynamics

## Abstract

Dimensions and surface properties are the predominant factors for the applications of nanofluidic devices. Here we use a thin liquid film as a nanochannel by inserting a gas bubble in a glass capillary, a technique we name bubble-based film nanofluidics. The height of the film nanochannel can be regulated by the Debye length and wettability, while the length independently changed by applied pressure. The film nanochannel behaves functionally identically to classical solid state nanochannels, as ion concentration polarizations. Furthermore, the film nanochannels can be used for label-free immunosensing, by principle of wettability change at the solid interface. The optimal sensitivity for the biotin-streptavidin reaction is two orders of magnitude higher than for the solid state nanochannel, suitable for a full range of electrolyte concentrations. We believe that the film nanochannel represents a class of nanofluidic devices that is of interest for fundamental studies and also can be widely applied, due to its reconfigurable dimensions, low cost, ease of fabrication and multiphase interfaces.

## Abstract (web summary)

Dimensions and the surface properties of the nanochannels are vital to the functions and applications of nano-fluidic devices. Here Ma et al. studied the film thickness of a Bretherton bubble in a microcapillary and demonstrate the liquid film can be used for label-free biosensing.

## Introduction

The application of micro- and nanofluidic devices has greatly boosted the development of the chemical and biological sciences and technologies^1–9^. When the typical length of a nanostructure approaches the thickness of the electrical double layer (EDL), some unique phenomena occur that have been widely used, for example for preconcentration^10^, desalination^11^, bio-sensing^12,13^, and energy conversion^14,15^. However, the technical barriers and cost of fabrication are considered to be constraints to the development of nanofluidics^16^. The invention of technologies helped to overcome these two barriers^17–21^. Besides, well-controlled dimensions and surfaces are critical for the physicochemical properties of nanofluidic devices. Deformable nanochannels could for example help in these respects, as the tunable dimension can be used for various applications. Recently, Bonhomme et al., reported a soft nanofluidic channel by using a foam bubble, which can produce a circular deformable nanochannel with minimum height down to 50 nm, using liquid/air interfaces instead of liquid/solid interfaces^22^. These interfaces introduce some specific properties like an electrical field dependent thickness^22^, concentration-independent conductance^23^, and an anomalous zeta potential^24^.

Inspired by the foam nanochannel^22^ and wetting films at surfaces^25,26^, here we report a film nanofluidic channel based on a thin layer of liquid created by inserting a gas bubble in a glass capillary. We name this technology bubble-based film nanofluidics (BFN). Although studies on liquid films surrounding bubbles in a capillary existed for decades^27–29^, the films were rarely developed as a nanofluidic device^30^, not speaking of a detailed characterization of their properties. Bubble-based film nanofluidic channels are fundamentally different from either solid-state nanochannels or foam nanochannels since here three (gas/liquid/solid) phases determine the properties instead of two phases. Compared to the solid-state nanochannel, the height of the film nanochannel can be tuned by changing the EDL thickness and wettability, with length of the film nanochannel tuned independently by applied pressure. Compared to the foam nanochannel, the film nanochannel can work in a surfactant-free environment, which is more friendly to biological samples^31^.

Here we demonstrate that the BFN can exhibit functionalities typical of solid state nanochannels, for instance ion concentration polarization (ICP), useful for pre-concentration and desalination. Besides, the film nanochannel performs even better than the solid-state nanochannel when used for label-free immunosensing, based on the principle of wettability change. We coated biotin on the capillary inner surface and demonstrated sensing of the biotin-avidin reaction in the film nanochannel. Our results indicate that the optimal sensitivity in the film nanochannel is two orders of magnitude higher than in the solid-state nanochannel, due to the change of wettability due to the reaction also influencing the film nanochannel properties instead of only a change in surface conduction as in the solid-state nanochannel. Thus, the immunosensing capabilities of the film nanochannel are sustained over a full range of salt concentrations, where in the solid-state nanochannel it only exists in low concentrated solutions.

## Results

### Devices and principles

**Fig. 1:**
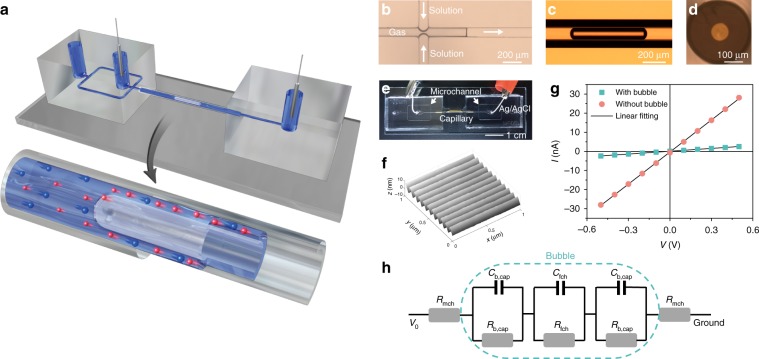
The principle of film nanochannel and setup. **a** Schematic of the principle. The film nanochannel was formed by inserting a gas bubble in a capillary. The film has length *L* and height *h*. Two connected electrodes were used for the electrical characterizations. **b** A snapshot of bubble formation within a PDMS microfluidic flow-focusing structure by high-speed camera (Photron, FASTCAM WX50). **c** A single bubble was conducted to a glass capillary and remained static in microscopic view, where it appeared circular from the cross-sectional view (**d**). **e** Picture of setup. Two electrodes were inserted in the pinched holes, and connected to a potentiostat for electrochemical measurements. **f** Wave-like patterns were found by AFM at the inner surface of capillary, creating an average roughness of 2.6 nm. **g** Typical *I–V* curves of a system with bubble (green) and without bubble (red). **h** An equivalent circuit of the system, consisting of resistances for the microchannel and film nanochannel and capacitances for the bubble meniscus (*C*_b,cap_, gas/liquid interface) and EDL of film nanochannel (*C*_fch_, liquid/solid interface).

We generated bubbles of equal size with a microfluidic flow-focusing structure, created by a standard PDMS chip fabrication and connection process (Fig. 1a)^32^. Nitrogen gas (99.9% pure) flows in the center channel (100 μm wide and 180 μm height), and was squeezed by the continuous flow of electrolyte solutions on the branch channels forming bubbles (Fig. 1b). The pressure of both the gas and water phases was kept constant at 20–50 mbar, operated by a pressure pump (Fluigent, MF CF-EZ).

The bubbles were then conducted from a rectangular PDMS channel to a circular capillary (Molex, 106815) of 100 μm inner diameter. We kept a single slug bubble in the capillary by stopping the flows, after which no apparent bubble shrinkage was observed for at least 12 h. The bubble in the capillary occupies most of the cross-sectional area of the glass capillary, leaving a cylindrical liquid film between solid and gas phase as shown in Fig. 1a. The Laplace pressure in the bubble works against the disjoining pressure in the liquid film^33^. The balance of the two pressures induces a specific film thickness that is determined by the EDL and the wettability of the solid surface. We characterized the film thickness by the electrical conductance using both Cyclic Voltammetry (CV) and electrochemical impedance spectrum (EIS) in a KCl solution, with two Ag/AgCl electrodes inserted at pinched holes in the PDMS chip (Fig. 1e). The details of chip fabrication and electrical characterizations are described in the Methods section. The liquid/gas interface is molecularly smooth due to the surface tension. Thus, the roughness of the capillary inner surface is critical to the properties of the film nanochannel. We characterized the roughness of the glass capillary inner surface by Atomic Force Microscope (Fig. 1f), and found an average roughness of 2.6 nm with length-oriented wave-like patterns, possibly originating from the capillary fabrication process^34^.

To derive the resistance of the film nanochannel, we used the equivalent circuit shown in Fig. 1h, which consists of the resistance of the microchannels including PDMS channels and capillary (*R*_mch_), of the film nanochannel (*R*_fch_) in case of a bubble residing in the capillary, and the capacitance from the gas/liquid interface at the bubble meniscus (*C*_b,cap_) and the EDL of the liquid film at the liquid/solid interface (*C*_fch_). The resistance from the microchannels can be experimentally characterized and also theoretically calculated when the dimensions and conductivity of liquids are known. The presence of a single bubble in the capillary significantly increased the system resistance as shown in Fig. 1g. This enabled us to derive the electrical resistance of the film nanochannel from the difference of resistance of the system with and without a bubble, *R*_fch_ = *R*_b,sys_−*R*_nb,sys_. Here *R*_b,sys_ and *R*_nb,sys_ represent the resistance of system with bubble and without bubble respectively. We could ignore the far smaller resistance of the microcapillary compared with the same length of the bubble (See Supplementary Note 1 for the calculations). The resistance induced by the bubble meniscus (*R*_b,cap_) can also be ignored compared to the film nanochannel (*R*_fch_) in our experiments, as we use a slug instead of a bubble (see Supplementary Note 2).

### Electrical characterization

**Fig. 2:**
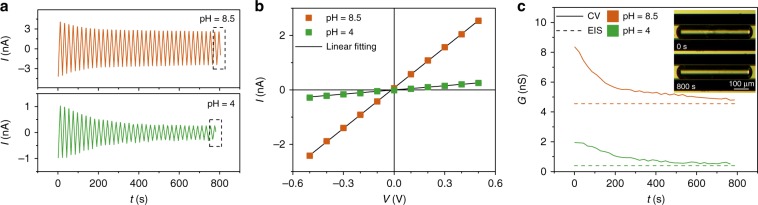
The electrical characterization of the film nanochannel. **a** The amplitude of film nanochannel conductance measured by cyclic voltammetry decreased in time, becoming saturated after a certain number of cycles, at pH values of 8.5 (top) and 4 (bottom), respectively. **b** Typical *I–V* curves at the saturated states at pH values of 8.5 and 4 (dashed), showing the Ohmic behavior of the system. **c** The evolution of system conductance measured by CV (solid lines) gradually reached the value measured by EIS (dashed lines). Inset figure shows snapshot of a single bubble before (top) and after (bottom) the electrical measurements, exhibiting a smoother surface after the measurements.

In our experiments, the typical bubble-length ranged from 0.5 to 1.5 mm, with the remainder of the microchannels filled with KCl solution. Figure 2a demonstrates typical CV cycles measured in 10 mM KCl at pH = 8.5 and 4. We found that the current amplitude gradually decreased and became saturated after ~10 cycles, finally dropping off to 60% of the first cycle at pH = 8.5. The decrease of conductance was more obvious in the acidic solution (pH = 4), with only 28% of conductance remaining compared to the first cycle. The reduction of conductance was found at the beginning of every electrical measurement after bubble generation, no matter how long we kept the bubble before applying electrical fields. However, once the conductance reached the saturated state, it remained constant. More details can be seen in Supplementary Note 3. Typical *I–V* curves of the saturated states with solutions at pH 8.5 and 4 demonstrated an Ohmic response to the applied voltage shown in Fig. 2b. By linear fitting the *I–V* curves (solid lines in Fig. 2b), we can derive the time evolution of the system conductance (Fig. 2c).

We performed EIS to characterize the conductance as a comparison to the conductance at the saturated states, as it avoids ion concentration polarization and limiting-current effects^12^. We found a similar conductance decrease in these EIS measurements, decreasing faster with higher voltage amplitude (see Supplementary Note 4). We found that the value measured by EIS at saturated state equaled the value measured by CV in the saturated state (Fig. 2c). More details are shown in Supplementary Note 5. We used the conductance values from EIS and the saturated states of CV in the further analysis, unless noted otherwise.

When trying to explain the conductance changes in CV, we noted that the surface of the liquid film appears smoother after 40 CV cycles (800 s) than at the start of the electrical measurement (see Inset of Fig. 2c). We hypothesize that initially a small volume of water was trapped within the liquid film, causing a higher conductance of the system. Also, contaminations could be trapped within the liquid film. We suspect that the reduction of conductance is due to the extraction of this trapped water by the electrical fields. The alternating electrical field generates electroosmotic flow transporting back and forth the trapped water and contaminants, and finally removing them from the liquid film, inducing the decrease of conductance. As reported, the side surface of a moving bubble is slightly concave due to the shear stress of fluids in the channel^35^. More details of our hypothesis and measurements can be found in the Supplementary Note 6.

Another point to be noted is that the bubble hardly moves in the capillary under the applied electrical fields in our experiments, except in case of a thick EDL at low salt concentration. However, the motion of the bubble in low concentration salt not necessarily induces a variation of film thickness. According to previous studies, the film thickness still remains constant when the capillary number (Ca) is <10^−4^ ^36–38^. The evidence from our electrical measurements also likely points to this conclusion (see Supplementary Note 7). In this paper, we focused in the experiments on the static or low Ca state (Ca is lower than 10^−6^, and the measured data can be seen in Supplementary Table 1) of the film nanochannel with a small amplitude of applied voltage by CV and EIS.

**Fig. 3:**
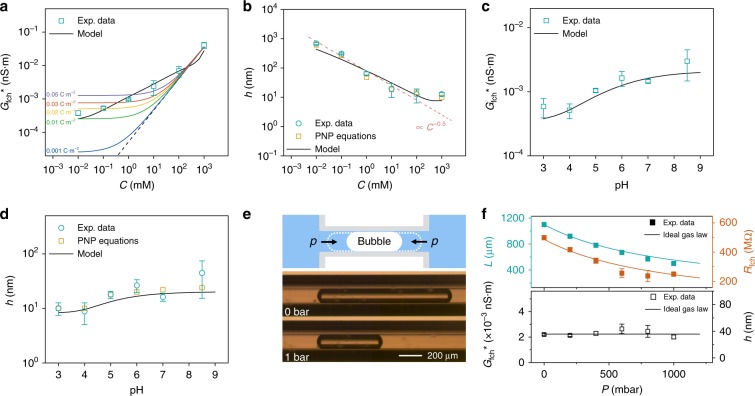
The tunable height and length of film nanochannel with characterizations. **a** The measured conductance of film nanochannel per unit length at pH 8.5 (dots), with theoretical predictions (black solid line). The colored lines are theoretical conductance of a fictitious solid-state nanochannel with different surface charge densities and a height of 11 nm. **b** The calculated film thickness from the normalized conductance as a function of KCl concentration at pH 8.5 by theoretical approach (circle dots) and simulations by PNP equations (square dots). Dashed line shows the dependence of the Debye length on concentration. **c** The normalized conductance with 10 mM KCl solution. **d** The calculated film thickness as a function of pH at 10 mM solutions. Solid lines are the theoretical predictions described in Methods section. **e** Bubble shrinkage by applied pressure. The length of the film channel decreases with increasing external pressure of liquid phase. Snapshot of a single gas bubble when the applied external pressure increases from 0 to 1 bar. The length decreases to nearly half value when 1 bar pressure applied on the microchannel. **f** The decrease of bubble-length (upper graph, green squares)-induced decrease of film nanochannel resistance (upper graph, orange squares) as a function of applied pressure. Solid lines were derived from Eq. 1 with length predicted by Ideal Gas Law. The normalized resistance of film nanochannel (lower graph, dots) and channel height (lower graph, dots) remain constant due to the persistence of capillary pressure (contact angle of bubble). The error bars are the standard deviation of five or more individual results.

We first investigated the effects of salt concentration and pH on the conductance, since changes in the EDL significantly influence the disjoining pressure, and hence the equilibrium film thickness. To eliminate the influence of differences in bubble length, we express the conductance of the film nanochannel per unit length, as *G*_fch_* = *L*·*G*_fch_. The displayed conductance is an averaged value with an error bar from at least five individual measurements (measured data can be seen in Supplementary Table 2 and 3). We calculated the error bars from five or more individual experiments using different capillaries, where the majority of errors originated from the variation of wettability at the capillary surface caused by the pre-cleaning process. A good cleaning or coating of the surface can help to increase the repeatability of film conditions as we demonstrated in immunosensing experiments (<4% error bars). Our experimental results (green dots in Fig. 3a) show that the conductance per unit length decreases by two orders of magnitude when the KCl solution is diluted from 1 M to 0.01 mM. Our theoretical prediction (black solid line) matches well with these experimental results. The dashed line in Fig. 3a represents the bulk conductance of a fictitious solid-state nanochannel with a fixed height of 11 nm. The system conductance is neither similar to that of a solid-state nanochannel shown with colored solid lines in Fig. 3a, which has a plateau at diluted concentrations dependent on the surface charge density^39^, nor to that of a foam nanochannel which has a conductance that remains of the same order at all concentrations^23^. As we will illustrate later in this paper, this results from the three-phase configuration of the film nanochannel with a concentration-dependent liquid film thickness.

From the measured conductance, we can calculate the film thickness by the following equation:

1 $$G_{{\mathrm{fch}}} = \frac{{2\pi rh}}{L}\kappa$$

where *r*, *h*, *L* are the radius of the capillary, the height of the nanochannel, and the length of the bubble, respectively. Conductivity has contributions of bulk solution conductivity *κ*_b_ and surface conductivity *κ*_s_, the latter originating from two different interfaces – liquid/gas and liquid/solid. The surface conductivity can be derived from the theory of electrokinetic flow in parallel-plate nanochannels and has the following form^40^:

2 $$\kappa _{\mathrm{s}} = \frac{{\mu \left( {\sigma _1 + \sigma _2} \right)}}{h}\left( {1 + \frac{1}{{2\pi l_{\mathrm{B}}\mu \eta }}} \right)$$

where *μ*, *l*_B_, *η*, *σ*_1_, and *σ*_2_ are the ionic mobility, Bjerrum length, liquid viscosity, and charge density of gas/liquid and solid/liquid interfaces respectively. The values of *σ*_1_ and *σ*_2_ were derived from previous studies^41–44^, and we experimentally characterized the zeta potential of the solid/liquid interface by measuring the streaming current in the capillary (see Supplementary Note 8; measured data can be seen in Supplementary Table 4). It needs to be noted that the accuracy of channel height estimation by Eq. 2 at low concentration is not as high as in high concentrations, as the surface conduction contribution can no longer be ignored in the diluted solutions. However, the height of film channel can still be estimated, since the conductance change induced by the height change is comparable to the contribution of the surface conductance in our low surface charge system. More detailed information can be found in Supplementary Note 9. The theoretical predictions demonstrate that the contribution from the surface conductivity depends on the film height for the entire range of salt concentrations. This is different from the solid-state nanochannel where the conductance reaches a plateau value due to the overlap of EDL where the conductance is becoming independent of channel height. In our film nanofluidic channel, the conductance change due to a change in film thickness can not be neglected, so that we could derive the film thickness by conductance measurements. Substituting Eq. 2 in 1, we obtain

3 $$h = \left[ {\frac{{G_{{\mathrm{fch}}}^ \ast }}{{2\pi r}} - \mu \left( {\sigma _1 + \sigma _2} \right)\left( {1 + \frac{1}{{2\pi l_{\mathrm{B}}\mu \eta }}} \right)} \right]/\kappa _b$$

Finally, substituting all physical constants (see Methods) and the measured conductance into Eq. 3, we can derive the height of the nanochannel shown as open circle dots in Fig. 3b. The height of film nanochannel can also be estimated by numerical simulations by Poisson-Nernst-Planck (PNP) equations, shown as open square dots in Fig. 3b. The estimated film height from both calculations and simulations did not represent a significant difference, and more details of the simulations can be found in Supplementary Note 9. Assuming the zeta potential remains constant in various concentrations, the Eq. 6 in Methods can be simplified as exp(**−***λh*) = *const*, where the ***λ*** is reciprocal of the Debye length. According to the definition of Debye length ***λ***^−1^ = (*ε*_0_*ε*_*r*_*k*_*B*_*T*/*e*^2^*CN*_A_)^1/2^ where *ε*_r_, *ε*_0_, *k*_B_, *T*, *e*, and *N*_A_ are relative permittivity of the liquid, electrical permittivity of vacuum, Boltzmann constant, temperature, elementary charge, and Avogadro constant, respectively, we know the film thickness is proportional to *C*^−0.5^ which is shown as the dashed line in Fig. 3b, matching well with our experimental results. The relationship between channel height and the normalized conductance of the film nanochannel can be seen in Supplementary Note 9. The theory (solid line) slightly deviates from the experimental data at diluted solution, due to the use of zeta potential at gas/liquid surface fitted from previous studies (See Supplementary Note 8)^41–43^.

As already briefly described in the device section, the thickness of the film nanochannel is determined by a balance between the disjoining pressure and the capillary pressure. The disjoining pressure at low salt concentrations (lower than 300 mM) is dominated by the long range electrostatic repulsion forces between the solid/liquid and gas/liquid interfaces, resulting in a high disjoining pressure and a thick film. The derived film nanochannel thickness ranges from 11 nm to 920 nm for salt concentrations from 1 M to 0.01 mM, well in accordance with the theoretical values. In high concentration solutions (higher than 300 mM), the short range van der Waals forces start to govern disjoining pressure and a minimal film thickness of 11 nm is reached. The thinnest film was in the range of common black films, due to the surface tension and wettability (CA equals to 25°) in our system^45^. In addition, the roughness of the capillary inner surface probably induces a thicker equivalent film.

The film thickness is also expected to be pH-dependent, as a higher pH value increases surface charge density and hence the electrostatic contribution to the disjoining pressure. We characterized the conductance of the film nanochannel per unit length for pH values ranging from 8.5 to 3, similar as above. The results in Fig. 3c demonstrate for a 10-mM KCl solution that the conductance decreases nearly an order of magnitude in this pH range, as well as the calculated channel height in Fig. 3d (32 nm to 10 nm). The lowest pH value is close to the isoelectric point of both glass surface (pH 2.8 ± 0.2^46^) and air/water surface (pH3.0 ± 0.5^42^), where both interfaces are nearly electroneutral.

Besides the height, the length of the film channel can be adjusted, as a compressible gas bubble is used. To keep the bubble in a static position in the capillary, we applied identical external pressures at the two ends of the microchannel (Fig. 3e). With applied pressures increasing from 0 to 1 bar, the bubble length gradually shrinks from 1100 µm to 500 µm (Fig. 3e), with the effects by pressure increase shown in Supplementary Note 10. The ideal gas law (*pV* = *nRT*, where *p*, *V*, *n*, *T, R* are the pressure, volume, the number of moles, thermodynamic temperature of ideal gas, and ideal gas constant respectively) well predicted the bubble-length decrease (black solid line in Fig. 3f).

The change of bubble length was observed by the increase of conductance as it is proportional to the bubble length according to Eq. 1. We found that the normalized conductance of the film nanochannel (*G*_fch_^*^) remained constant at varying external pressure, indicating a constant height of the film nanochannel (Fig. 3f). This is in accordance with the equation in our theoretical predictions in the Methods section. The film thickness is determined by the equilibrium of disjoining pressure and capillary pressure, the latter of which relates to the contact angle of the bubble that remained constant. The constant capillary pressure causes a constant film thickness even when the absolute pressures in both gas and liquid phase increase.

Summarizing, our results demonstrated that the dimensions—both height and length—of the film nanochannel are widely and independently tunable, the height by adjusting salt concentration or pH and the length by applying external pressure. A reconfigurable dimension of film nanochannel may not only help to decrease the costs and technical barriers of making nanofluidic devices by avoiding the necessity of fabricating numerous nanofluidic devices with different geometries for specific uses. As we will demonstrate later in this paper, the film nanochannels also can be operated as another type of highly sensitive (bio)chemical sensors, using the property of wettability.

### ICP in film nanochannel

Nanofluidic channels can generate ion concentration polarization (ICP) due to their ion permselectivity, generally allowing predominantly cations to pass^11,47^. ICP causes salt accumulation and depletion at the two opposite ends of the nanochannel during prolonged passage of current. The ICP phenomenon is useful in biomolecule pre-concentration, seawater desalination, and more^11,48,49^. Here we demonstrate that the film nanochannels also give rise to ICP.

**Fig. 4:**
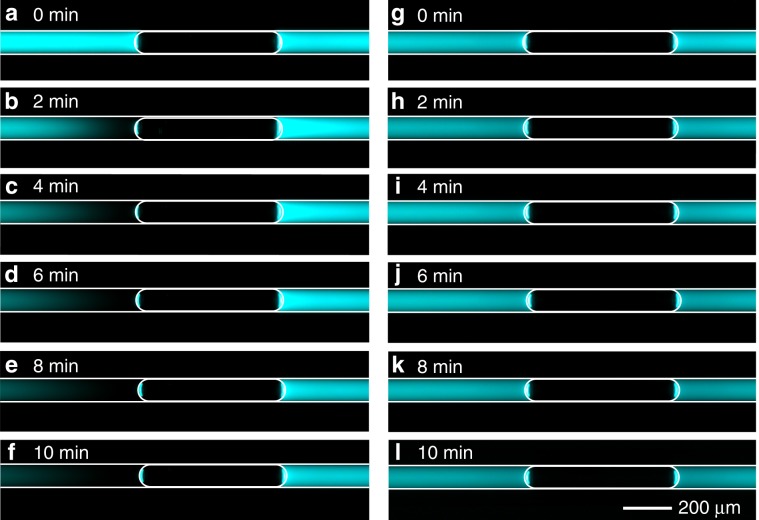
Ion concentration polarization by film nanochannel. **a**–**f** ICP effects were observed in 70 μM STB solution, while not in 3 mM STB solution (**g**–**l**), with 30 μM fluorescein used as fluorescence dye. The images were recorded every 2 min, to prevent the photobleaching of fluorescence. White solid lines were drawn to outline the capillary and the position of the bubble.

Two different concentrations (70 µM and 3 mM) of sodium tetraborate buffer solution at pH 8 (STB, Na_2_B_4_O_7_) were used in the experiments, with addition of 30 μM fluorescein as fluorescent concentration indicator. Depletion of fluorescein (dark) was readily observed in 70 µM STB solution after applying 10 V DC voltage for 2 min (Fig. 4b), and became more obvious over time (Fig. 4c–f), while this phenomenon was not observed in the high concentration STB solution (Fig. 4g–l). Fluorescein enrichment was not obvious on the opposite side of the bubble, probably due to photobleaching by the continuous UV excitation^50^. The recorded conduction current under DC applied voltage shows a continuous decrease and finally saturation for diluted electrolyte solution, while nearly remaining constant at high concentration solutions (see Supplementary Note 11). The current-limiting effects at low salt concentration can be attributed to the high resistance of the depletion zone created.

### Label-free immunosensing by BFN

**Fig. 5:**
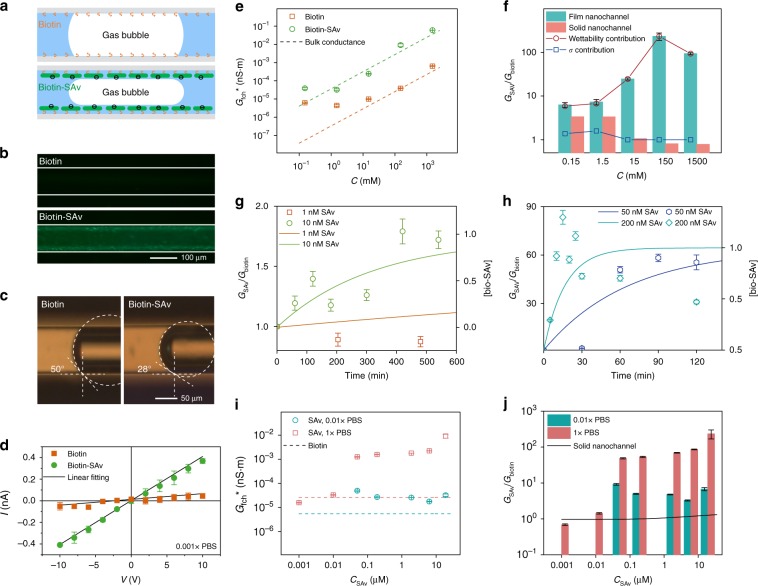
Label-free immunosensing by film nanochannel. **a** Schematic picture illustrating the principle of sensing the biotin-SAv reaction. **b** The functionalization with biotin (top) and subsequent successful binding of avidin on the capillary surface (bottom) was demonstrated by the fluorescence of FITC-SAv. **c** A change of contact angle in the capillary was observed after the immobilization of SAv. **d** The *I–V* curves show a strong change of the conductance induced by the biotin-SAv binding reaction (19 μM SAv). **e** The bubble length-normalized conductance of the film nanochannel *G*_fch_^*^ was derived by linear fitting in 10^−3^× to 10× PBS solutions. The dashed lines are the theoretical conductance of a ‘fictitious’ solid-state nanochannel neglecting the surface conductance. **f** Sensitivity (*G*_SAv_/*G*_bio_) of film nanochannels (green) as function of salt concentration, compared to that of a polysilicon nanochannel^13^ (orange column). The blue square dots represent the contribution from the change of zeta potential, while the red circular dots represent the contribution from the wettability change. **g** The kinetics of the biotin-SAv reaction in 1 nM (orange square dots) and 10 nM (green circle dots) SAv solutions, with predicted ratio (0 to 1) of occupied biotin sites (right axis, solid lines). **h** The reaction kinetics in 50 nM (dark blue dots) and 200 nM (light blue dots) SAv solutions, with predicted ratio of biotin-SAv binding (right axis, solid lines). **i** The bubble-length normalized film nanochannel conductance *G*_fch_^*^ as a function of SAv concentration for 0.01× and 1× PBS solutions. The dashed lines represent the measured conductance before biotin-SAv binding at 0.01× (blue) and 1× (red) PBS, while the dots are measured *G*_fch_^*^ after SAv binding. **j** Calculated sensitivity as a function of SAv concentrations in 0.01× and 1× PBS solutions. The solid line represents data from a solid-state nanochannel^51^. The error bars are the standard deviation obtained from five or more individual measurements.

Nanofluidic channels have been used for biosensing using the principle of surface conductance (the conductance in the EDL), because a (bio)chemical binding reaction such as the biotin-avidin binding^13,51–53^ causes a change of surface charge density, which causes a conductance change in diluted solution (Fig. 5a)^13^.

We will use the sensitivity of a conductivity-based immunosensor as a critical performance indicator, which we define as the ratio of the conductance after *G*_SAv_ and before *G*_biotin_ the SAv reaction:

$${\mathrm{Sensitivity}} = G_{{\mathrm{SAv}}}/G_{{\mathrm{biotin}}}$$

We will demonstrate that the sensitivity of immunosensing in a film nanochannel is enhanced due to the change of wettability in addition to changes in surface charge density.

We coated the inner surface of the capillary by applying a self-synthesized PLL-g-OEG-Biotin^54,55^ solution to the capillary inner surface. The cationic macromolecule Poly-l-Lysine (PLL) assembled on the negatively charged glass capillary surface, and oligo ethylene glycol (OEG) was used to block non-specific binding^54^. Successful functionalization of the capillary with biotin and reactivity of the immobilized biotin was demonstrated using subsequent binding of fluorescein isothiocyanate-labeled Streptavidin within 1–2 h as microscopically observed (Fig. 5b). We then pumped liquids through the capillary for the surface coating (Biotin) and binding reaction (SAv), and only then generated a single bubble in the capillary to form a film nanochannel for the measurement of conductance. (see Methods) Although pressure-driven flow was used to accelerate the binding reactions, our system was still operated in the mass transport-limited regime, with a Damkohler number (*D*_a_) of 70.

We found a clear change of the contact angle, before (CA is ~50°) and after the specific SAv reaction (CA is ~28°), probably caused by the increase of surface charge density after binding (Fig. 5c). The increased wettability on SAv binding decreases the contact angle and importantly also increases the disjoining pressure and film thickness (see the theory in Methods section).

We characterized the conductance of the biotin-coated film nanochannel by CV and EIS in phosphate-buffered saline (PBS) solution, to obtain the conductance changes caused by the Biotin-Avidin reaction. The *I–V* curves at 0.001× PBS demonstrate an obvious conductance change after SAv binding (Fig. 5d), enabling to derive the conductance by linear fitting of *I–V* curves. We found that the film conductance at 0.001× PBS increased by nearly an order of magnitude, which is higher than the conductance increase found in a solid-state nanochannel (1.2–3.4)^13,51^. We attribute this to a major factor of wettability change, and a minor factor of surface conductance change, as we will discuss below. We repeated the conductance characterization after biotin-SAv reaction at various PBS concentrations (Fig. 5e). The error bars in Fig. 5 were calculated from the repeated electrical measurements from a single capillary with five or more generated bubbles. The errors of <4% demonstrate the reliability of the electrical detection. The conductance behaves similar to the bulk solution conductance of a solid-state nanochannel, since a fictitious conductance (dashed line) of a solid-state nanochannel matched well to our results. The equivalent height of the nanochannel after SAv reaction is 7 nm (green), while the equivalent height is only 0.07 nm (red) before the SAv reaction, indicating that a stable liquid film at the biotin-coated surface did not exist.

The sensitivity as immunosensor was defined by the ratio of the conductance after and before the SAv reaction: *G*_SAv_/*G*_bio_, as a critical performance indicator. Figure 5f shows that the sensitivity of the film nanochannel is higher than reported in the data for solid-state nanochannels in diluted solution, including conductance change (approximates to 4)^13^. Moreover and surprisingly, we found that the sensitivity of the film nanochannel is even higher in the high concentration solutions, with a maximal sensitivity of 233 in 1× PBS solution.

To illustrate the mechanism of sensitivity enhancement and effects of the salt concentration, we separated the contributions to the sensitivity from the surface conduction and wettability. We derived the contribution from the surface conduction using the zeta potential of the biotin and SAv surface^13^, as shown in Supplementary Note 12. We found that the sensitivity due to surface conductance (square blue dots in Fig. 5f) behaved similarly to that in the solid-state nanochannel but at nearly half the value, since only the solid/liquid interface can be coated and functional for the sensing. By subtracting the contribution of surface conduction, we can find that the contribution from the change of wettability (circular red dots in Fig. 5f) is predominant.

In diluted solutions, the contribution of the surface conductance can no longer be ignored. However, the sensitivity caused by the surface conductance change is weaker than by the wettability change as can be seen from the sensitivity of solid state nanochannels^13^, since the surface conductance linearly responds to the change of surface charge density (*κ*_s_ is proportional to *σ*)^51^. As the surface conduction plays a more important role in diluted solutions, the system sensitivity decreases and approaches that of the solid-state nanochannel.

In concentrated solutions, the conduction can be estimated as ***G***_fch_^*^~2*πhκ*_b_∝*h*, where the height dominates the conductance. For a solid state nanochannel the height remains constant, hence it will not present a conductance variation after reaction. Consequently, solid states nanochannels cannot be used for biosensing at high salt concentration when using the principle of surface conductance change. However, in a film nanochannel where both wettability and surface charge determine the thickness of the wetting film, conductance can be used for biosensing. The immobilization of the SAv molecules results in a change of both wettability and surface charge density. A well-wetting surface results in a small contact angle and large Laplace pressure, and a thick liquid film, which was described in Supplementary Eq. 16 in Supplementary Note 12. The critical influence of the wettability can be seen by the results of pH effects as a control group (Fig. 3c, d). The normalized conductance only increased by one order of magnitude for a pH change from 3 (electroneutral surface) to 8.5 under a constant CA (25°). This change, due to the increase of surface charge density, is much smaller than the change observed in the immunosensing experiment. Summarizing, due to the contributions of both wettability and surface conduction, the label-free sensing of biotin-SAv reaction can be electrically measured over a full range of salt concentrations in the film nanochannel.

To investigate the minimum concentration and binding kinetics of SAv, we measured the conductance change of the liquid film performing the binding reaction in a 1× PBS solution in time. We hereby followed the procedure Reactions and measurements in the Methods section. We found a minimum detectable SAv concentration of 10 nM, with a sensitivity equal to 1.2–1.7 due to the small change of CA (Fig. 5g). Unfortunately, lower concentrations (lower than 10 nM) of SAv could not be detected, probably due to mass transport limitations in the microcapillary, where the Damkohler number was 70 ≫ 1 ($D_{\mathrm{a}} = \frac{{k_{\mathrm{a}}\left[ {{\mathrm{biotin}}} \right]\delta }}{{D_{{\mathrm{SAv}}}}}$, where *k*_a_, [biotin], *δ*, *D*_SAv_ are association rate constant, surface density of biotin, thickness of depletion layer and diffusion coefficient of SAv, respectively). Here the Damkohler number *D*_a_ represents the ratio of reaction rate and diffusive mass transport rate. The mass transport rate determines the binding rate at *D*_a_ >1, while the reaction rate is dominating at *D*_a_ < 1. To further decrease the minimum detectable concentrations, working in a finer capillary will be helpful to increase mass transport.

At concentrations above 50 nM, a change of CA could already be microscopically observed, inducing a sensitivity of 50–60 (Fig. 5h). Our results show that binding can already be measured at an early stage by the liquid film nanochannel. For example, for 200 nM SAv the change of CA saturated at a sensitivity of 60 within 10 mins. Thus not only the sensitivity is enhanced as compared to solid-state nanochannels, but also the speed of sensing is increased. This possibly indicated a nonlinear response of the binding kinetics to the micro-bubble contact angle change in the capillary. Although the real-time characterizations of the SAv binding ratio at the capillary inner surface is technically not easy in our current device, it would be interesting to investigate the connections between binding kinetics and contact angle change in the future work. The decrease of sensitivity in 200 nM SAv was possibly caused by the damage to the biotin film at the capillary surface by the hydrodynamic shear stress of the pressure-driven flow. A similar phenomenon was found in previous work in solid-state nanochannels^51^. More details about our theoretical model can be found in Supplementary Note 13.

Finally, by using the film nanochannel, we could quantify the minimum detectable SAv concentration at the equilibrium states, except the SAv concentrations lower than 10 nM. We took the conductance at concentration of 1 and 10 nM SAv after 10 h binding reactions in Fig. 5i, since a longer reaction is not useful in the clinical diagnostic. To separately demonstrate the effects from wettability and surface conduction, we chose two concentrations of PBS solution – 0.01× PBS and 1× PBS, where the former measurements are expected to be determined by changes in surface conduction and the latter by wettability changes.

To perform the binding reactions, SAv solutions with concentrations ranging from 1 nM to 19 μM were used, following the procedure in Reactions and measurements. Figure 5i shows the normalized conductance of the film channel after biotin modification (dashed lines) and SAv binding (dots) for the two PBS concentrations. The normalized conductance after SAv binding significantly increased and remains at the same order of magnitude as they reached the equilibrium states when the SAv concentration is over 50 nM.

Using our definition, we calculated the sensitivity as a function of SAv concentration at 0.01× PBS and 1× PBS (Fig. 5j). Compared to the sensitivity in solid state nanochannels (solid line derived from a previous study^51^), the sensitivity of the film nanochannel is higher for both diluted and concentrated solutions. For diluted solutions, the wettability-induced height change contribution is comparable to that of the surface conduction, thus increasing the sensitivity of the film nanochannel. For high concentration solutions, the height change influence is predominant, with the sensitivity reaching the magnitude of 100 at 1× PBS. Finally, the film nanochannel can be useful for the electrical measurement of binding equilibria for immunosensing. Some further minor factors determining the sensitivity of the film nanochannel are discussed in Supplementary Note 14.

Our results demonstrate that the film nanochannel has great promise for label-free biosensing, with a maximum sensitivity that is two orders of magnitude higher than in a solid state nanochannel. Besides, the biosensing works over the entire common range of salt concentrations (0.1 mM to 1000 mM), with the optimal working condition actually at the physiological conditions of a 1× PBS solution. By using the mechanism of CA change, the binding can be measured at an early stage before equilibrium binding is reached, accelerating the speed of sensing. These results indicate that the wetting-based sensing allows working at the physiological salt concentrations, which can be very useful for clinical diagnostics. Furthermore, film nanochannels are easy-to-make, low cost, and as demonstrated possess a number of unique properties which are attractive for further development and study.

## Discussion

We reported a liquid film nanochannel produced by inserting a gas bubble in a cylindrical glass capillary. We electrically characterized the film conductance, and calculated the film thickness. We found that the height and length of the film nanochannel can be individually tuned by EDL thickness and applied pressure, which is important for making dimension-reconfigurable nanochannels for non-specific uses. We demonstrated that the film nanochannels share some fundamental properties with other nanofluidic devices, such as ion concentration polarization. Finally, we demonstrated that the film nanochannel can be used as another type of label-free biosensor using the principle of wettability change. With the biotin-SAv binding reaction as model, our results indicate that the maximal sensitivity of the film nanochannel can be two orders of magnitude higher than for a solid state nanochannel. This surprising result can be explained by a binding reaction-induced change of surface wettability that causes a large change of film thickness. In addition, the binding can be measured at an early stage before the equilibrium state is reached, accelerating the speed of sensing. The optimal working concentration is that of physiological solution (1× PBS), opening up attractive possibilities for clinical analytical applications.

## Methods

### Device fabrication

The PDMS chip was fabricated by standard photolithography processes. A layer of 180 μm negative photoresist (Microchem, SU-8 2075) was spin-coated on a polished silicon wafer. The photoresist was then exposed using a mask and mask aligner with 24 mW cm^−2^ UV light density for 15 s. After developing the microstructures for 5 min, we obtained a SU8 mold with 180 μm height microchannels. PDMS was mixed with curing agent at a ratio of 10:1, poured on the lithography wafer and baked at 60 °C for 4 h. The PDMS chip was then fabricated by standard PDMS—PDMS bonding technique using an oxygen plasma cleaner (Harrick Plasma, PDC-002). A channel structure of 360 μm width was designed for capillary connection. After inserting the capillary into the PDMS channel, some uncured PDMS was applied at the junction of PDMS and capillary, to ensure water tightness after curing.

Hydrogen peroxide (30% H_2_O_2_) and diluted potassium hydroxide (0.1 M KOH) were sequentially injected for 15 and 45 min respectively, as a pretreatment of the capillary surface to have a clean and well wettable surface. Then, the solutions prepared for experiments were flushed through the capillary for at least 45 min before the electrical measurements, to obtain an equilibrium state of the surface chemistry, especially when working at different pH solutions.

The cleaning of the capillary can be repeated per 48 h ensuring the wetting state of the capillary. The capillary can be reused for many times by a drying process (100 °C for 0.5 h) and cleaning procedure, unless becoming contaminated or physically breaking down.

### Solution preparation

The solutions used for conductance measurements were prepared by first dissolving the monovalent salt KCl (from Sigma-Aldrich) at 1 M in DI water (Merck Millipore, D24 UV). The solutions of other concentrations were prepared by diluting this solution. The pH value of the solution was adjusted with diluted KOH and HCl, avoiding other ionic species introduced in the electrolyte solution. The solutions were re-prepared every 24 h to avoid the absorption of CO_2_. The solution pH was characterized with a pH meter (LeiCi, PHS-25), while the conductivity of the solution was characterized with a conductivity meter (Mettler Toledo, FE38). All experiments were performed at room temperature (22 °C).

### Electrical characterization

The gas bubble was generated by operating a computer-controlled pressure pump while monitoring under a Microscope (Zeiss, Axio Observer A1). When one bubble had entered the capillary, the remainder of the gas bubbles were removed from the PDMS channels, ensuring one single bubble remaining. Homemade Ag/AgCl electrodes were used to minimize overpotentials of the electrochemical reactions. They were connected to a pico-ammeter voltage source station (Keithley 6482) for CV characterizations controlled by in-house LabVIEW software, and an electrochemical workstation (CHI660E) for EIS measurements. The CV characterizations were operated using a triangular wave voltage with scanning rate of 0.1 V s^−1^ and amplitude of 0.5 V for at least 800 s. As a control experiment, we operated the EIS characterizations at 0.5 V amplitude of voltage with frequencies ranging from 1 to 1k Hz immediately after the CV.

### Biotin and SAv preparation

PLL (15–30 kDa) and FITC-SAv were purchased from Sigma-Aldrich. EZ-Link *N*-hydroxysuccinimidyl ester NHS-OEG4-Biotin was purchased from Thermo SCIENTIFIC. Streptavidin was purchased from J&K Scientific. PLL-g-OEG4-Biotin was synthesized by adding NHS-OEG4-Biotin into a 40-mg mL^−1^ solution of PLL dissolved in 50 mM Na_2_CO_3_, and reacting for 5 h at room temperature.

### Reactions and measurements

We first pumped the self-synthesized PLL-g-OEG-Biotin through a microcapillary for a homogeneous coating of Biotin on its inner surface for at least 2 h. Then we pumped specific concentrations of SAv solutions in for the binding reactions, without bubble insertion. Only then, we generate a single bubble with specific PBS solutions in the capillary, to form the liquid film nanochannel for the electrical measurements.

### Surface modification

The capillary was first rinsed with 0.1× PBS (pH 7.2; 10× PBS contains 1.55 M KCl, 0.015 M KH_2_PO_4_, and 0.027 M K_2_HPO_4_) for 2 h, then immersed in a 1-mg mL^−1^ solution of biotin in 10 mM 4-(2-hydroxyethyl)-1-piperazineethanesulfonic acid (HEPES) for 1 h to complete the surface modification with biotin. The capillary was then immersed for 1 h in a 200-nM FITC-SAv solution (also in 10 mM HEPES) to verify the modification result of biotin and to test the specific binding. A 0.1× PBS solution with 1 mg mL^−1^ SAv (19 μM), was used to research the effect of specific binding on channel conductance, immersing the capillary for 10 h. Other SAv solutions of different concentration were diluted from 1 mg mL^−1^ stock solution, and immersion time still kept as 10 h. All the experiments were performed at room temperature.

### Theoretical predictions

The thickness of the liquid film, as previously reported, results from an equilibrium between capillary pressure ***p***_c_ and disjoining pressure Π_d_, expressed as^56^:

4 $${\Pi}_{{\mathrm{d}}} = p_{{\mathrm{c}}} = P_{{\mathrm{g}}} - P_{{\mathrm{ext}}} + \frac{{\gamma {\mathrm{cos}}\theta }}{r}$$

where *P*_g_ and *P*_ext_ are the gas pressure inside the bubble and the external pressure. Furthermore, *γ*, *r*, *θ* are interfacial tension, inner radius of capillary, and wetting angle, respectively. As the pressure difference between inside and outside of the bubble is determined by the meniscus curvature, we can derive the capillary pressure as $p_{{\mathrm{c}}} = \frac{{\gamma {\mathrm{cos}}\theta }}{r}$. The contact angle of the bubble can be optically characterized in the capillary. (See Supplementary Note 15). The disjoining pressure is in general seen as composed of two terms, one representing van der Waals forces and the other electrostatic interaction Π_d_ = Π_vdW_ + Π_el_, where the van der Waals pressure resulting from molecular interactions can be calculated as^57^

5 $${\Pi}_{{\mathrm{vdW}}} = - \frac{A}{{6\pi h^3}}$$

Here *A* is the Hamaker constant of the medium in the liquid film, and *h* indicates its thickness. The electrostatic repulsion can be calculated as:

6 $${\Pi}_{{\mathrm{el}}} = 64kTC_\infty \gamma _1\gamma _2{\mathrm{exp}}\left( { - \lambda h} \right)$$

7 $$\gamma _{{\mathrm{i}}} = \tanh \left( {\frac{{ze\zeta _{{\mathrm{i}}}}}{{4kT}}} \right),{{\mathrm{i}}} = 1,2$$

Here *k*, *T*, *C*_∞_, 1/*λ*, *h*, *e*, and *z* are the Boltzmann constant, temperature, bulk number density of the electrolyte ions, Debye length, film thickness, elementary charge, and ionic valence, respectively. *γ*_1_, *γ*_2_ are the reduced surface potentials of the liquid/air and liquid/solid interfaces, with corresponding zeta potentials *ζ*_1_, *ζ*_2_ respectively. Since it is still nearly impossible to characterize the liquid/solid surface potential^58^, here we took the zeta potential instead of the surface potential to estimate the thickness of the liquid film. According to the Supplementary Eq. 17, this deviation causes a negligible difference on the calculated liquid film. Since the characterization of zeta potential was performed when using pure gas bubbles without surfactants, the measured value might already take the slippage into account. Here we did not take additional slippage effects into account in the calculations. Finally, we can derive the theoretical value of liquid film thickness, as well as the predicted film resistance shown in Fig. 3. It needs to be noted that the theory described above is only valid for static liquid films or low Ca number.

### Physical constants

*μ*: ionic mobility, 8.1 × 10^−9^ m^2^ s^−1^ V^−1^

*η*: liquid viscosity, 1.01 × 10^−3^ Pa s

*l*_B_: Bjerrum length, 0.7 nm

*γ*: interfacial tension, 7.2 × 10^−2^ N m^−1^

*e*: elementary charge, 1.6 × 10^−19^ C

*A*: Hamaker constant, −1.03 × 10^−21^ J

*k*: Boltzmann constant, 1.38 × 10^−23^ J K^−1^

*T*: temperature, 295.15 K

*k*_a_: association rate constant, 5 × 10^6^ M^−1^ s^−1^

[biotin]: surface density of biotin, 5.5 × 10^−8^ mol m^−2^

*δ*: thickness of depletion layer, 1.5 × 10^−5^ m

*D*_SAv_: diffusion coefficient of SAv, 6 × 10^−11^ m^2^ s^−1^

## Supplementary information

Supplementary Information

Description of Additional Supplementary Files

Supplementary Movie 1

## Data Availability

The data that support the plots within this paper and other finding of this study are available from the corresponding author upon reasonable request.
