# Supplementary material for: Dimension-reconfigurable bubble film nanochannel for wetting based sensing
Source: Nat Commun. 2020 Feb 10;11:814. doi: 10.1038/s41467-020-14580-x (PMC7010761; doi:10.1038/s41467-020-14580-x)
Supplement: Supplementary file 1 — Supplementary Information [file 41467_2020_14580_MOESM1_ESM.pdf]

Supplementary Information to "Dimension-Reconfigurable bubble film nanochannel for wetting based sensing"

Ma *et al*.

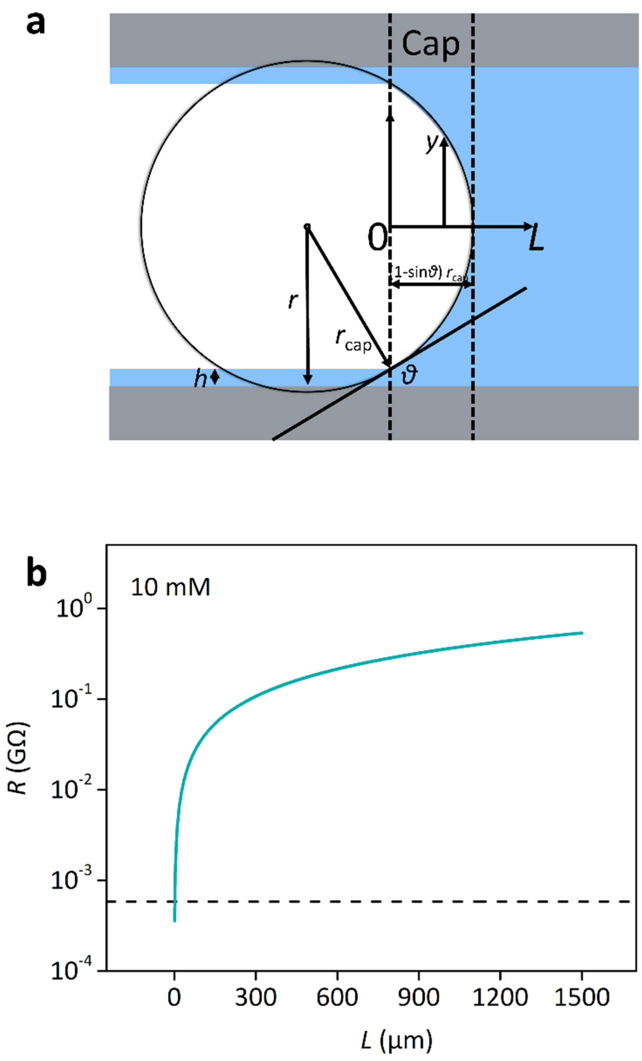

Supplementary Figure 1. Approximate calculation of cap resistance compared to the film nanochannel resistance. a, The calculation principle and geometric relationship of bubble cap. **b,** The resistance of bubble in the capillary as a function of length (solid line) compared with the bubble cap resistance (dashed line).

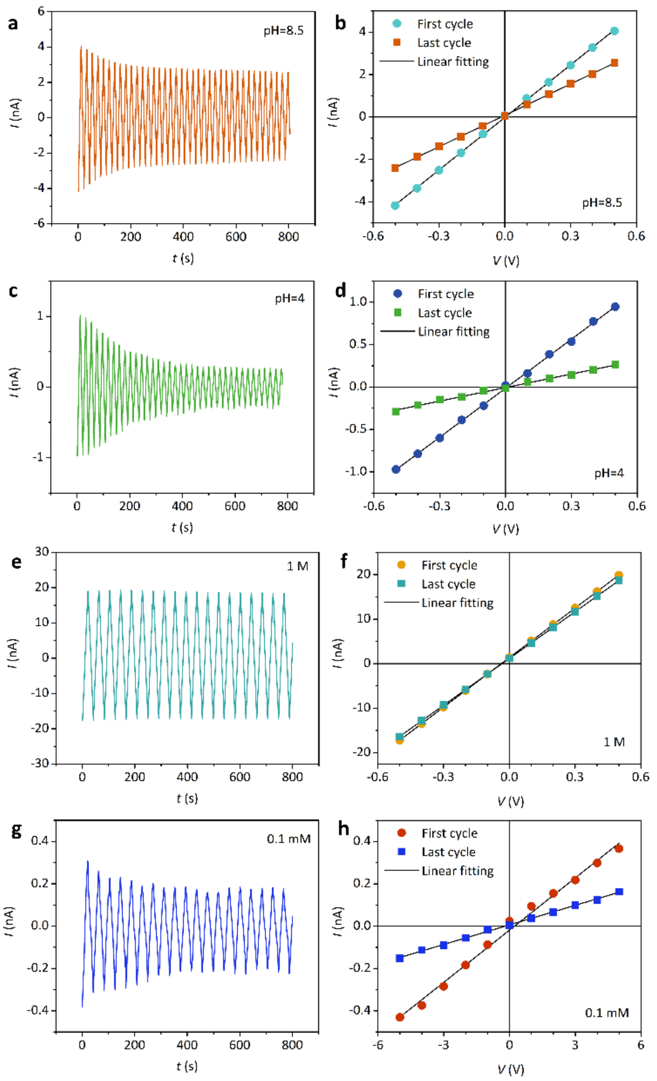

Supplementary Figure 2. The current curves changing with time and the typical I-V curves of first cycle and last cycle of CV measurement. a, c, e, and **g** reflects the saturated state in all experiments conditions. **b,** The amplitude of current decreased 40% before getting saturated in KCl solution of 10 mM and pH = 8.5; **d,** the current drop was 72% for 10 mM and pH = 4; **f,** the current drop was 6% for 1 M and pH = 8.5; **h,** the current drop was 63% for 0.1 mM and pH = 8.5. Solid lines are derived from linear fitting.

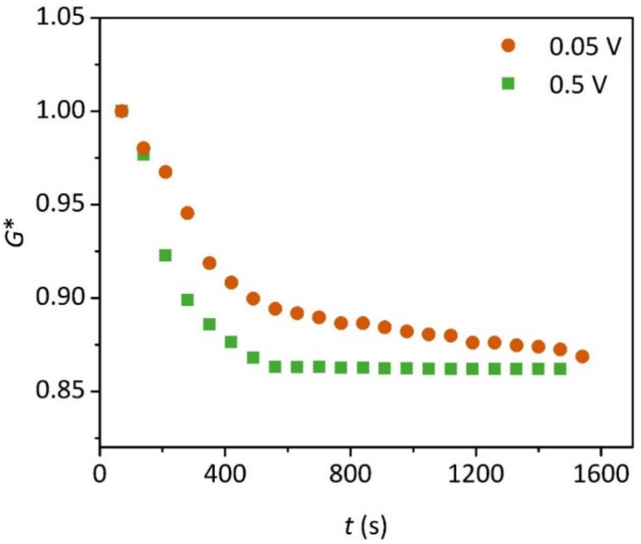

Supplementary Figure 3. The conductance of nanochannel in 10 mM KCl at pH = 8.5 measured as time step. The film impedance gradually decreased and became saturated after about 550 s under 0.5 V bias voltage (green square dots), while took over 1600 s to reach the saturated state under 0.05 V voltage (orange circle dots).

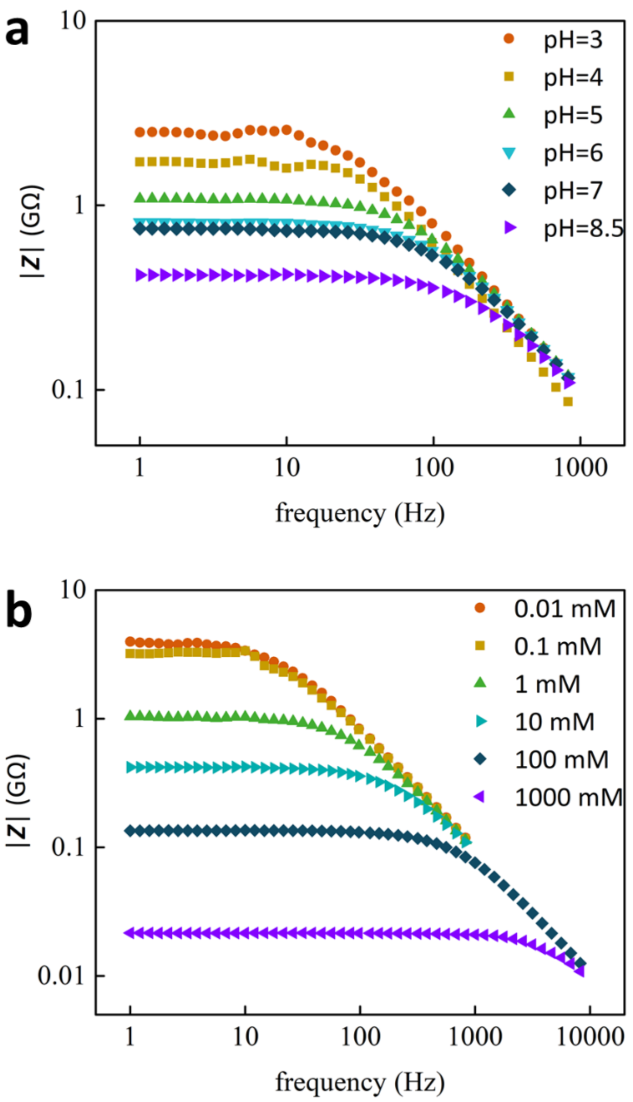

Supplementary Figure 4. The impedance of the film nanochannel characterized in different pH and concentration solutions. a, The experimental data of the normalized nanochannel impedance derived in 10 mM KCl for pH values ranging from 8.5 to 3. **b,** The experimental data of the normalized nanochannel impedance derived in pH 8.5 KCl solutions ranging from 1 M to 0.01 mM.

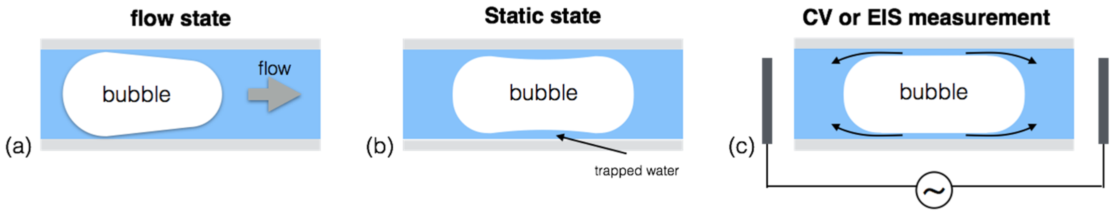

Supplementary Figure 5. The hypothesis to explain the conductance drop. The thickness of the EDLs are related with channel height and drainage in the film when applying voltage.

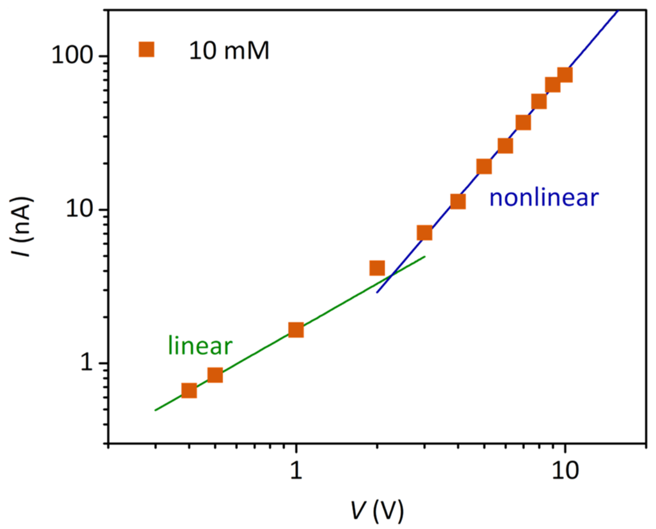

Supplementary Figure 6. The current measured when applying different voltages. The DC voltage was varied from 0.4 V to 10 V. The current could be separated in a linear region and a nonlinear region. We focused on the linear region in our research, where the film channel height remained stable.

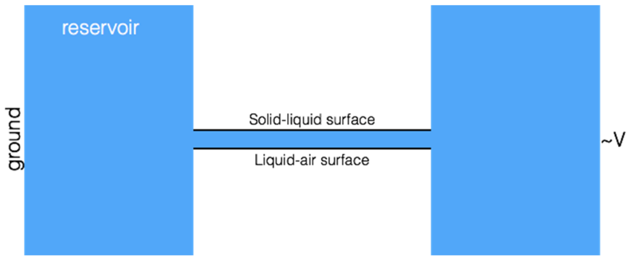

Supplementary Figure 7. Scheme of simulation model. A 1D parallel-plate nanochannel was inserted between reservoirs containing KCl electrolyte solution. The inner surfaces of the nanochannel, corresponding to the solid/liquid and liquid/gas interface of the film nanochannel, can be individually charged according to the experimental results.

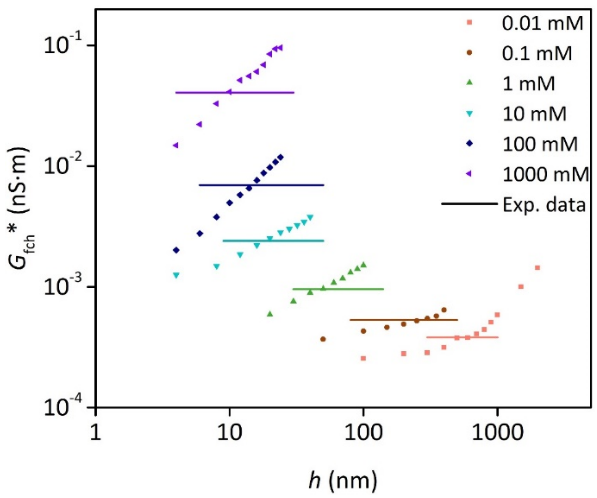

Supplementary Figure 8. The nanochannel conductance variation with film thickness in solutions ranging from 0.01 mM to 1 M. Simulation results (dots) reflect the conductance changes with channel height, due to low surface charge density in our bubble film nanochannel.

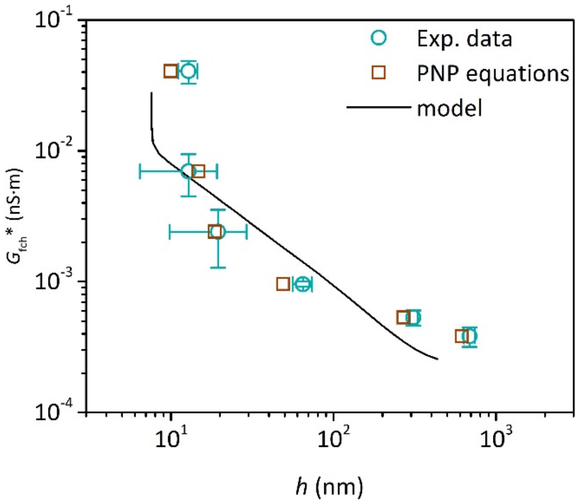

Supplementary Figure 9. The film conductance variation with the height of film channel. The normalized conductance of film nanochannel at pH 8.5 (green circle dots) was derived from experiments, and compared with simulation results (orange square dots) and theoretical predictions (black solid line). The channel height was calculated according to Equation 3.

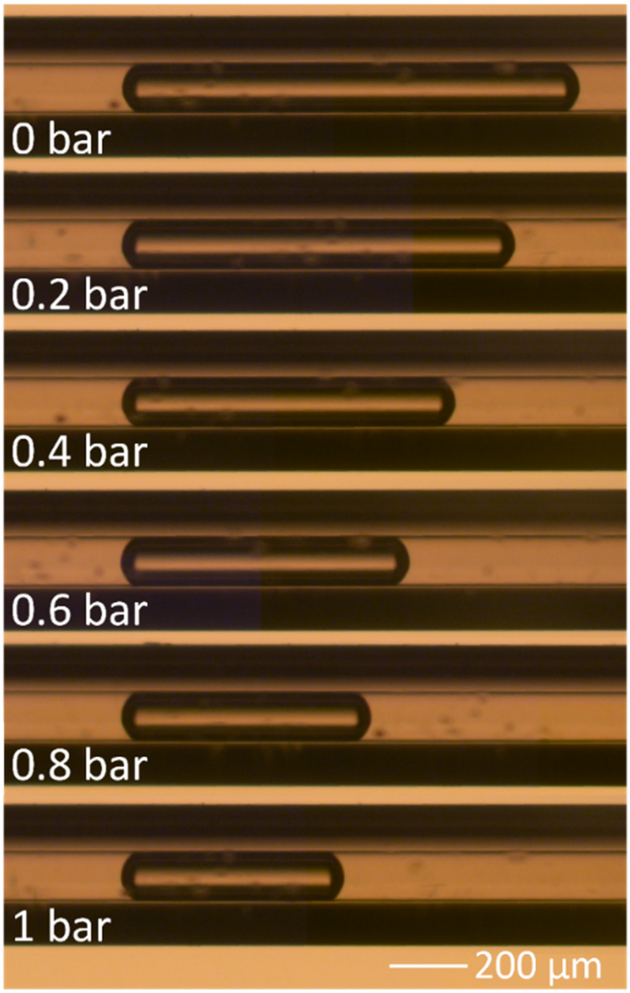

Supplementary Figure 10. Snapshot of gas bubble when increasing the pressure at both ends of bubble. The bubble was compressed from the initial 0 bar to 1 bar, recording a micrograph after steps of 0.2 bar.

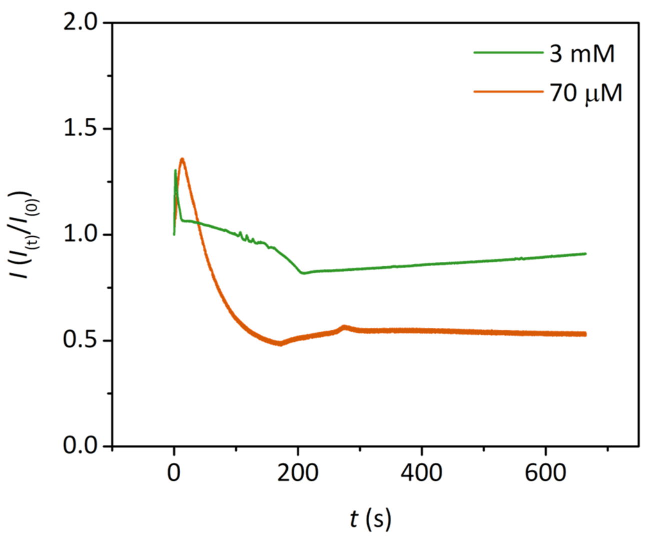

Supplementary Figure 11. The variation of the normalized current in the film nanochannel when the ICP effects arose. The current measured in 70 µM STB solution (orange line) decreased to 52% of its initial value after applying 10 V DC voltage for 600 s, while the current remains at 91% of its initial value in 3 mM STB solution.

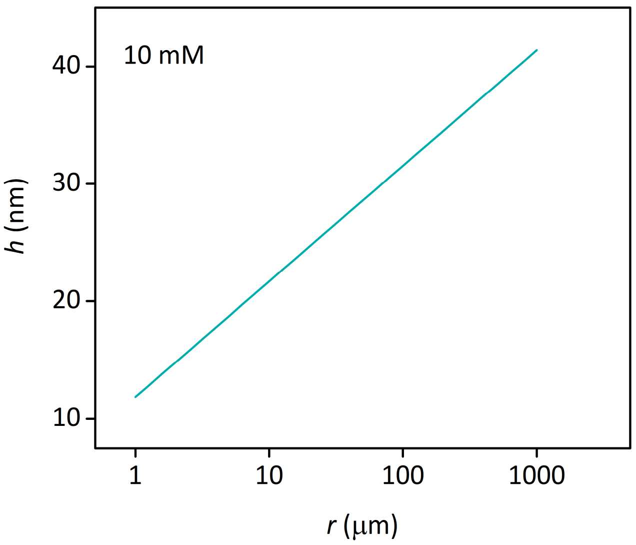

Supplementary Figure 12. The height of the film nanochannel as a function of capillary inner radius. When in 10 mM KCl solution, the film thickness increases from 12 nm to 41 nm with the capillary radius increasing from 1 µm to 1000 µm.

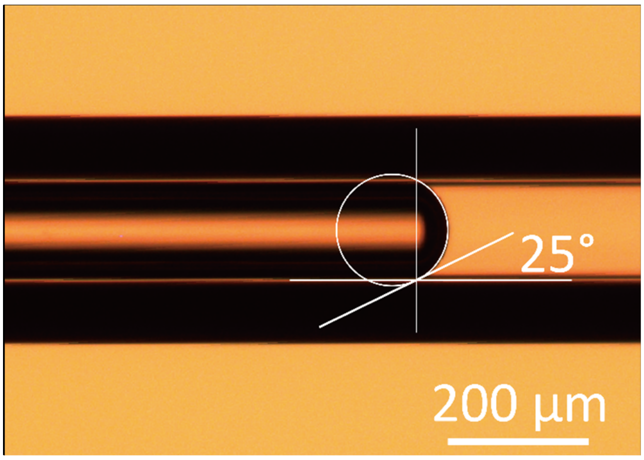

Supplementary Figure 13. The optical micrograph of the bubble in the capillary. The contact angle was measured with image processing software by standard measuring methods. A circle was used to fit the curvature of the bubble cap, and the slope angle of the tangent line is taken as the bubble contact angle.

Supplementary Table 1. The mean velocity of bubble and Ca in different concentration solutions when applying 0.5 V DC voltage. The pH of all solutions is 8.5.

***C* (mM)**	**0.01**	**0.1**	**1**	**10**	**100**	**1000**
***U* (mm/s)**	0.0214	0.0136	0.0193	0.0143	0	0
**Ca**	2.97e-7	1.89e-7	2.68e-7	1.98e-7	0	0

The velocity of bubble and Ca in different pH solutions when applying 0.5 V DC voltage. The concentration of all solutions is 10 mM.

**pH**	**3**	**4**	**5**	**6**	**7.4**	**8.5**
***U* (mm/s)**	7.30e-4	2.43e-3	3.16e-3	6.37e-3	0.0150	0.0143
**Ca**	1.02e-8	3.39e-8	4.40e-8	8.86e-8	2.09e-7	1.98e-7

Supplementary Table 2. The measurement data of film nanochannel conductance (nS·m) in different concentration solutions. The pH of all solutions is 8.5.

Index	1	2	3	4	5
**0.01 mM**	3.10e-4	3.04e-4	3.71e-4	4.47e-4	4.47e-4
**0.1 mM**	5.95e-4	5.92e-4	5.95e-4	4.63e-4	4.77e-4
**1 mM**	1.01e-3	9.72e-4	9.94e-4	9.10e-4	9.39e-4
**10 mM**	1.79e-3	1.67e-3	1.78e-3	2.61e-3	2.18e-3
**100 mM**	5.49e-3	4.44e-3	6.35e-3	9.89e-3	8.01e-3
**1000 mM**	5.33e-2	4.64e-2	3.42e-2	4.06e-2	3.75e-2

Supplementary Table 3. The measurement data of film nanochannel conductance (nS·m) in different pH solutions. The concentration of all solutions is 10 mM.

Index	1	2	3	4	5
**8.5**	2.62e-3	4.67e-3	1.70e-3	2.35e-3	3.31e-3
**7**	1.56e-3	1.44e-3	1.38e-3	1.20e-3	1.49e-3
**6**	1.95e-3	1.82e-3	1.15e-3	1.30e-3	1.64e-3
**5**	1.06e-3	9.76e-4	1.10e-3	1.04e-3	9.91e-4
**4**	5.02e-4	3.85e-4	6.52e-4	5.76e-4	4.04e-4
**3**	7.33e-4	6.70e-04	3.64e-4	6.35e-4	4.59e-4

Supplementary Table 4. The zeta potential of gas/liquid and solid/liquid interface. The pH of all solutions is 8.5.

***C* (mM)**	**0.01**	**0.1**	**1**	**10**	**100**
**ζ_{gl} (mV)**	-57.0	-42.1	-28.9	-27.4	-17.3
**ζ_{sl} (mV)**	-117	-115	-94.3	-65.0	-35.9

The zeta potential of gas/liquid and solid/liquid interface. The concentration of all solutions is 10 mM.

**pH**	**3**	**4**	**5**	**6**	**7**	**8.5**
**ζ_{gl} (mV)**	-0.633	-1.51	-6.21	-10.8	-16.8	-27.4
**ζ_{sl} (mV)**	-5.37	-14.0	-25.6	-41.9	-53.7	-65.9

Supplementary Table 5. The contact angle before and after biotin-streptavidin reaction. There was no obvious correlation between contact angle and concentration of PBS solution.

***C* (mM)**	**0.15**	**1.5**	**15**	**150**	**1500**
**Biotin**	48°	45°	45°	48°	42°
**Biotin-SAv**	28°	26°	26°	27°	26°

Supplementary Table 6. The height of nanochannel derived by experiments and simulations in different concentration solutions.

C (mM)	0.01	0.1	1	10	100	1000
**Our theory (nm)**	687	310	65	20	13	13
**Simulation (nm)**	615	269	49	19	15	10

Supplementary Note 1. The resistance of micro-capillary compared to film nanochannel.

Here we derive under what conditions the resistance of the micro-capillary (R_c) can be ignored compared to the resistance of a liquid film (R_{fch}) of the same length (L), $R_c \ll R_{fch}$. According to the definition of capillary and film nanochannel resistance:

$$R_c = \frac{1}{\kappa_b}\frac{L}{\pi r^2} \quad (1)$$

$$R_{fch} = \frac{1}{\kappa}\frac{L}{2\pi r h} \quad (2)$$

Hence, we can take Supplementary Equation 1 and 2 into the above assumption ($R_c \ll R_{fch}$) as follows:

$$\frac{1}{\kappa_b}\frac{L}{\pi r^2} \ll \frac{1}{\kappa}\frac{L}{2\pi r h} \quad (3)$$

We find that the R_c can be ignored when the height of the film nanochannel is much smaller than the capillary radius $h \ll r/2$. According to the previous studies of bubbles in a capillary, under the condition of low capillary number or static, film thickness is typically less than 1% of the capillary radius[1]. Thus, we can directly use the system resistance difference with and without bubble, to derive the resistance of the liquid film, ignoring the resistance of the capillary channel with length L.

Supplementary Note 2. The resistance of cap $R_{\text{b, cap}}$ compared to that of the film nanochannel.

Here we derive when the resistance from the bubble meniscus can be ignored compared to the resistance of the film nanochannel, in case of our experiments. We first assume the direction of electric field in the cap is along the axis of the capillary, and thus calculate the resistance of the cap approximately by

$$R_{\text{b,cap}} = \frac{L}{\kappa S} \tag{4}$$

where S is the cross-sectional area of the annular cap. The conductivity (κ) consists of contributions of bulk conductivity κ_{b} and surface conductivityκ_{s}. $\kappa = \kappa_{\text{b}} + \kappa_{\text{s}}$, $\kappa_{\text{s}} = \frac{\mu(\sigma_1+\sigma_2)}{r-y}\left(1 + \frac{1}{2\pi l_{\text{B}}\mu\eta}\right)$, where r is the radius of the capillary. Other parameters have been labelled in Supplementary Figure 1a, or can be obtained from our manuscript. Then we can deduce the equations below from the geometrical relationships:

$$S = \pi(r^2 - y^2) \tag{5}$$

$$r = r_{\text{cap}} \cos\theta + h \tag{6}$$

$$y = \sqrt{r_{\text{cap}}^2 - \left(r_{\text{cap}} \sin\theta + L\right)^2} \tag{7}$$

Finally, by taking all the equations and infinitesimal method, we can rewrite the resistance of bubble cap as the following equation:

$$R_{\text{b,cap}} = \int \frac{2dL}{\kappa S} = \int_0^{r_{\text{cap}}(1-\sin\theta)} \frac{2dL}{\pi\kappa(r^2 - y^2)}$$

$$= \int_0^{r_{\text{cap}}(1-\sin\theta)} \frac{2dL}{\pi\left(\kappa_{\text{b}} + 1.67\dfrac{\mu(\sigma_1+\sigma_2)}{r - \sqrt{r_{\text{cap}}^2 - \left(r_{\text{cap}}\sin\theta + L\right)^2}}\right)\left[r^2 - r_{\text{cap}}^2 + \left(r_{\text{cap}}\sin\theta + L\right)^2\right]}$$

$$= \int_{r_{\text{cap}}\sin\theta}^{r_{\text{cap}}} \frac{2dL}{\pi\left(\kappa_{\text{b}} + 1.67\frac{\mu(\sigma_1+\sigma_2)}{r-\sqrt{r_{\text{cap}}^2 - L^2}}\right)\left(r^2 - r_{\text{cap}}^2 + L^2\right)} \tag{8}$$

Hence, we derived the resistance of the bubble cap in 10 mM KCl solution by integral calculation (Supplementary Figure 1b). The solid line reflects the resistance of the film nanochannel as a function of channel length, while the dashed line represents the total cap resistance of a bubble. For a bubble from 500 μm to 1500 μm, the film resistance is 2 to 3 orders of magnitude larger than the bubble cap resistance, which indicates the rationality of ignoring the bubble cap resistance in the resistance calculation.

Supplementary Note 3. The decrease of conductance during CV measurements.

A decrease of conductance always occurred in our CV experiments, after the bubble generation and from the beginning of every electrical measurement. Here we show the results of CV measurements over time, and the typical Ohmic response after the first and after the last cycle shown on the right side correspondingly in Supplementary Figure 2. Our results demonstrate that the current and resistance after a large number of cycles becomes saturated. However, the decreases of current seem relevant to the electrolyte strength and surface charge density.

Supplementary Note 4. The decrease of conductance during EIS measurements.

We characterized the nanochannel impedance by EIS as time steps until it reached a saturated value, for two different voltage amplitudes (0.5 V and 0.05 V) from 1 Hz to 1000 Hz. The results from EIS also demonstrated that the conductance gradually decreases with the number of cycles (Supplementary Figure 3). We suspect the reduction of conductance might be induced by electroosmotic flow which drives trapped water out of the liquid film under the electrical field. We found the reduction of conductance under 0.5 V to be faster than under 0.05 V amplitude.

Supplementary Note 5. EIS characterization of the film nanochannel.

EIS was performed at the saturated state, and was operated at 0.5 V voltage amplitude at frequencies ranging from 1 Hz to 1k Hz. Ag/AgCl electrodes were used for the measurement in the same manner as for the CV characterization. The results in Supplementary Figure 4a demonstrate for a 10 mM KCl solution that the impedance increases nearly an order of magnitude in the pH range of 3 to 8.5, while it increases by over two orders of magnitude when the solution is diluted from 1 M to 0.01 mM (Supplementary Figure 4b).

Supplementary Note 6. The hypothesis of conductance decrease.

Here we briefly explain our hypothesis on the reduction of conductance during the electrical measurements.

As described in our manuscript, a small volume of water was trapped in the film channel during the dynamic state of bubble (Supplementary Figure 5a). The trapped water will be kept inside the film due to the equivalent pressure difference between the two caps of the bubble, unless external forces like the applied electric force drive out the water. (Supplementary Figure 5b)

The applied electrical field induces an electrical current and electroosmotic flow due to the coupling of water molecules and ions in combination with the surface charge on the capillary inner surface and at the gas/liquid interface. The electroosmotic flow induces the flow of water out of the film. The pressure at the bubble center becomes identical to the pressure at the cap, thus avoiding water flow in the liquid film again. (Supplementary Figure 5c) The removal of trapped water causes a decrease of the conductance, finally reaching a stable value.

Supplementary Note 7. The film conductance under applied E-fields.

The bubble will hardly move during electrical measurements, unless adding surfactants or in highly diluted solutions. The motion of the bubble does not necessarily induce a thicker liquid film. The previous experiments demonstrated that the film thickness still remains constant when the capillary number of bubble motion is less than 10^{-4} [1-3], which likely has also been proved by our electrical measurements. Here we took 10 mM electrolyte solution as an example, under various applied voltages (Supplementary Figure 6). We found that the current responds linearly to the voltage when the applied voltage is below 1 V, however starts to increase above 1 V. This possibly indicates that the height of the film nanochannel remains constant below 1 V while becoming thicker due to viscous entrainment of moving liquid above 1V, instead of reflecting the balance of disjoining pressure and capillary pressure[4]. This matches the theoretical predictions by Teletzke et al. theory[5] and Hodges et al.[6]. In this paper, we probed the static state (or low Ca regime) of the nanochannel by using a small applied voltage.

Supplementary Note 8. Zeta potential at the solid/liquid and liquid/gas interface.

Here we characterized the zeta potential of the solid/liquid interface by the streaming current in the capillary. When we apply an external pressure difference between the two ends of the capillary, the counterions in the electrical double layer at the water/glass interface move with the liquid, which represents a streaming current, determined by zeta potential ζ as follows[7]:

$$I_{\mathrm{str}} = -\frac{\varepsilon_{\mathrm{r}}\varepsilon_0 \pi r^2}{\eta}\frac{\Delta P}{L}\zeta \tag{9}$$

where ε_{r}, ε_0, r, ΔP, η, L are relative permittivity of the liquid, electrical permittivity of vacuum, capillary radius, applied pressure difference, liquid viscosity and capillary length.

The measurement of streaming current was performed using a pump with a constant applied pressure. The KCl solution was pumped through the capillary, and we measured the current in the capillary as the streaming current at the water/glass interface and calculated the zeta potentials.

We derive the zeta potential of the gas/liquid interface according to the method in the literature of Aref Seyyed Najafi *et al.*[8]. The zeta potential was obtained by measuring the electrophoresis of bubbles in the electrolyte solution. The solutions to be measured were immersed in ice-water mixture to keep the temperature at 0 °C, and at the same time, nitrogen was continuously injected into the solution for 12 hours to obtain a saturated solution of nitrogen. The solutions were rapidly heated up to room temperature (22 °C) by using a heater, and then the bubble electrophoretic velocity was measured with a zetasizer (MALVERN, ZEN3600). The zeta potential of bubbles could be calculated and given automatically by the instrument.

In order to calculate the zeta potential in a convenient manner, we fitted the function of zeta potential ζ(mV) according to the data we collected in previous studies and experiments[9-12]. The fitting functions relating to pH (Supplementary Equation 10) and salt concentration C (mM, Supplementary Equation 11) are shown below.
For the solid/liquid interface

$$\zeta = -74.1 + \frac{1.85+74.1}{1+10^{-0.403\times(5.43-\mathrm{pH})}} \tag{10}$$

$$\zeta = 14.9 \times \ln(C + 0.420) - 104 \tag{11}$$

and for the gas/liquid interface as a function of pH (Supplementary Equation 12) and C (mM, Supplementary Equation 13):

$$\zeta = -\frac{31.0\times 4.12\times 10^{-6}\times \mathrm{pH}^{6.49}}{1+4.12\times 10^{-6}\times \mathrm{pH}^{6.49}} \tag{12}$$

$$\zeta = 4.34 \times \ln(C - 0.0019) - 45.2 \tag{13}$$

Supplementary Note 9. Film conductance G_{fch}^* vs the channel height *h*.

Here the height of the film nanochannel is a derived value from the measured conductance. The minimal film thickness calculated from the conductance is 11 nm, slightly different from the theoretical predictions. This difference might be caused by the wave-like patterns at the capillary inner surface as we demonstrated in Fig. 1f. Water within these wave-like patterns induces additional conductance and an equivalently thicker film on average.

We performed simulations based on the PNP equations and calculated the conductance of a nanochannel with a certain surface charge and a variable height in COMSOL 5.3. The model geometry was a 1D nanochannel of infinite width and a height varying from 5 nm to 2 μm (shown as Supplementary Figure 7). This model can be applied to our liquid film since we have a closed circular nanochannel with width (circumference of capillary) of over 300 μm which is at least 2 to 3 orders of magnitude larger than the height. The reservoir was filled by the corresponding solution, and the surface charge density of both interfaces was set as the value we derived from the measured zeta potentials by the Grahame equation. In the simulation, we imposed no-slip boundary conditions at the interfaces, although the zeta potential that we obtained from Zetasizer characterization (shown in Supplementary Note 8) may have been enhanced by slip at the gas/liquid interface. The simulation model is illustrated below.

The conductance of nanochannel was governed by Poisson-Nernst−Planck equations showing below:

$$\mathbf{J}_\mathrm{i} = -D_\mathrm{i}\left(\nabla C_\mathrm{i} + \frac{F}{RT} Z_\mathrm{i} C_\mathrm{i} \nabla V\right) \tag{14}$$

$$\nabla(\mathbf{J}_\mathrm{i} + C_\mathrm{i}\mathbf{u}) = 0 \tag{15}$$

$$\nabla^2 \phi = -\frac{F}{\varepsilon} \sum_\mathrm{i} Z_\mathrm{i} C_\mathrm{i} \tag{16}$$

where J_i, D_i, C_i, Z_i are the current flux, diffusion coefficient, concentration and valence of ionic species i, respectively. The ε, u, F, R, T, V, ϕ are the dielectric constant of the liquid, liquid velocity, Faraday constant, gas constant, temperature, applied voltage and surface potential, respectively.

The conductance of the nanopore/channel was calculated by $G = I/V$, and $I = \int_0^{T_0} 2\pi r\,(J_\mathrm{i} + \rho u_\mathrm{v})dr$, where ρ and u_v are the space charge density and the electroosmotic velocity, respectively. Thus we can derive the normalized conductance of the nanochannel as a function of height at different concentrations (Supplementary Figure 8). We then can determine the channel height by matching the conductance at a specific height with the measured values. This estimated channel height was then inserted together with our approximate theory by Equation 2 in Supplementary Figure 9. The data from experiments and simulations are also shown in Supplementary Table 6 for comparing.

Supplementary Note 10. Tunable length of the film nanochannel.

The length of the film nanochannel is tunable due to the compressibility of the gas bubble (as shown in Supplementary Figure 10), which length is inversely proportional to the applied pressure according to the ideal gas law.

Supplementary Note 11. The current recordings under ICP effects.

The current decreases during the observation of the ICP phenomenon. The prominent ICP effects in 70 μM STB solution can be seen by current measurements in Supplementary Figure 11.

Supplementary Note 12. The contribution of surface conduction to the sensitivity of the immunosensing.

As expressed by Equation 4 in the theoretical predictions section, the force equilibrium in the liquid film is between capillary pressure and disjoining pressure as $p_c = \Pi_{el}$, where the Van der Waals force has been ignored. Hence, the relationship between surface charge and film thickness can be calculated as:

$$64kTC_{\infty}\gamma_1\gamma_2 exp(-\lambda h) = \frac{\gamma\cos\theta}{r} \tag{17}$$

where γ_2 corresponds to the reduced surface potentials of the liquid/solid interface, which changes when the reaction of biotin and SAv occurs. We can write Supplementary Equation 17 in the following form:

$$h \sim \ln(B_1\gamma_2) \tag{18}$$

where $B_1 = \frac{r64kTC_{\infty}\gamma_1}{\gamma\cos\theta}$ is a constant when just considering the change of liquid/solid interfacial charge induced by SAv binding. Actually, the immobilization of SAv also changes CA, and the film thickness and θ have the relationship as:

$$h \sim B_2 + \ln(|\gamma_2|/\cos\theta) \tag{19}$$

where $B_2 = \ln(|B_1|\cos\theta)$ is independent on the liquid/solid interface charge and CA. The film thickness is inversely proportional to the change of contact angle, so that a well-wetting surface with small contact angle induces a thick film.

Thus the sensitivity that results from surface conduction changes (binding of SAv) can be expressed as:

$$\frac{G_{\mathrm{SAv,s}}}{G_{\mathrm{bio}}} = \frac{h_{\mathrm{SAv,s}}}{h_{\mathrm{bio}}}\frac{\kappa_{\mathrm{SAv}}}{\kappa_{\mathrm{bio}}} = \frac{\ln(B_1\gamma_2')}{\ln(B_1\gamma_2)}B_3 \tag{20}$$

where $B_3 = \frac{\kappa_{\mathrm{SAv}}}{\kappa_{\mathrm{bio}}}$ is also a constant, and γ_2' represents the reduced surface potential after the biotin – SAv reaction. As the change of surface charge only occurred at the liquid/solid interface, the sensitivity contributed by surface conduction in the film nanochannel is lower than in the solid-state nanochannel.

Supplementary Note 13. The adsorption kinetics of biotin-SAv binding.

The theoretical model of binding kinetics shown in Fig. 5f-h was derived according to the Langmuir adsorption model by the following equation[13]:

$$[\mathrm{bio-SAv}] = \frac{[\mathrm{SAv}]}{[\mathrm{SAv}]+K_\mathrm{D}} \cdot (1 - \exp[-t(k_\mathrm{a}[\mathrm{SAv}] + k_\mathrm{d})]) \qquad (21)$$

where [SAv], k_a, k_d are concentration of SAv, association rate constant, and dissociation rate constant, respectively. Equilibrium dissociation constant represents the ratio of dissociation rate constant and association rate constant $K_D = k_d/k_a$. The degree of adsorption of the biotin-SAv specific reaction ranges between 0 and 1, and the reaction equilibrium $\frac{[\mathrm{SAv}]}{[\mathrm{SAv}]+K_\mathrm{D}}$ will be reached after sufficient reaction time.

Supplementary Note 14. Height change as a function of capillary radius.

The film thickness can be expressed as a function of capillary inner radius according to Equation 4 in our manuscript. The capillary radius is proportional to the capillary pressure as $p_{\mathrm{c}} = \frac{\gamma \cos\theta}{r}$, thus we can derive the relationship between channel height and capillary radius from Supplementary Equation 17 as:

$$h = \frac{1}{\lambda} \ln Br \qquad (22)$$

where B is a constant and equal to $B = \frac{64kTC_{\infty}\gamma_1\gamma_2}{\gamma \cos\theta}$. The relationship curve is shown in Supplementary Figure 12.

Supplementary Note 15. The characterization of contact angle in the micro-capillary.

The snapshot of the bubble was recorded with the CCD camera of the microscope. The bubble shape was fitted with Drop Shape Analysis (DSA). This image analysis software fitted the bubble contour with a circle. This circle model intersected with the base line on the substrate (glass), where the gradient of the circle was the contact angle of the bubble on the glass (Supplementary Figure 13).

We found that the contact angle has an obvious decrease after the binding reaction with Avidin, independent of the salt concentration (Supplementary Table 5). This again demonstrated that the principle of sensing is caused by the contact angle, which induces a transition from a non-wetting surface (no stable film existence) before binding to a well-wetting surface (stable film) after binding.

Supplementary Discussion: Minor effects on Sensitivity

Surface roughness and sensitivity

Here, we discuss some minor factors relevant for the biosensing performance of the film nanochannel. As we demonstrated, the wetting properties of the capillary inner surface determine the formation of the liquid film and therefore the sensitivity. Thus, a wide-range transition of contact angle is helpful to further increase the sensitivity. Previous studies have demonstrated that the wettability can be affected by the surface roughness[14]. For instance, a hydrophobic surface may transit into a super-hydrophobic surface by an appropriate micro-nano structuring. The sensitivity can thus potentially be further enhanced when the capillary inner surface can transit from a super-hydrophobic state where no conductive path exists along the micro-bubble, to a hydrophilic state with an apparent thick liquid film on an immunoreaction. However, a roughened surface may not always work for increasing the sensitivity. If the change of wettability is not large enough to achieve an appropriate hydrophobic state, the conductive path will be dominated by the roughened surface instead of the film, thus inducing a smaller change of conductance and sensitivity.

Radius of capillary and sensitivity

A bubble in a small radius capillary results in a thin film, due to the high capillary pressure. With Equation 4 we can calculate the film thickness as a function of capillary radius, assuming the other physical conditions remain the same. Supplementary Note 10 shows that the film thickness increases from 12 nm to 41 nm when the capillary radius increases from 1 μm to 1000 μm at 10 mM KCl solution. As can be seen, the effect of the capillary radius on the film thickness is negligible compared to that of the other surface properties like wettability, EDL and surface charge density. Since the normalized conductance can be approximated by $G_{\mathrm{fch}}{}^{*} \sim 2\pi r h$, the sensitivity of the film nanochannel is determined by the height (h) change, independent of radius (r). From the analysis above, the capillary radius has minor effects on the height change, and thus is a minor factor for the sensitivity. However, the use of a bubble in a nano-capillary is not recommended, as the high Laplace pressure will lead to gas dissolution in the aqueous solution.

Influence of the homogeneity of the film nanochannel

Liquid film thicknesses are typically characterized by reflectometry. However, this is not feasible for our film nanochannel, since the bubble meniscus is too small for the point light source to focus on the film surface. Moreover, the glass capillary reflects the light several times leading to erroneous values of the film thickness. The film thickness calculated from the electrical conductance measurement results in an averaged value. As we described above, the wave-like rough surface may induce inhomogeneity of the channel height, although the gas-liquid phase is likely to remain smooth. An atomically smooth solid surface may thus be helpful for a homogeneous thickness of the film nanochannel, and future applications. Furthermore, a well pretreated surface is

important to obtain a homogeneous wettability and consequentially a homogeneous film thickness.

Slip Boundary at liquid-solid interface

The slippage may occur at low concentration of surfactant of liquid/vapor interface by simulations[15], which is a comparable case in our system without using surfactants. According the results, the zeta potential could be enhanced with a slip boundary. Here we characterized the zeta potential according to the method in literatures[8], and take as the zeta potential of liquid/vapor interface without taking additional slip boundary effects. The theoretical predictions using the characterized zeta potential matched well with our experimental results, which possibly indicates the characterized values of zeta potential we used are close to the real situation.

However, this doesn't indicate there is no slip at liquid/vapor interface, since the characterized zeta potential by using microbubbles may already include the effects of slippage. In such case, we considered the slippage of the liquid/vapor interface by using the corresponding zeta potentials, for the calculations of liquid film. The existing of slippage even by AC electrical field with small bias voltage (EIS measurement) might induce the overestimation of film channel height, however need further studies and design of experiments.

Supplementary References

1. Chen, J. Measuring the film thickness surrounding a bubble inside a capillary. *J. Colloid Interface Sci.* **109**, 341-349 (1986).
2. Bretherton, F. P. The motion of long bubbles in tubes. *J. Fluid Mech.* **10**, 166-188 (1961).
3. Chaudhury, K., Acharya, P. V. & Chakraborty, S. Influence of disjoining pressure on the dynamics of steadily moving long bubbles inside narrow cylindrical capillaries. *Phys. Rev. E* **89**, 053002 (2014).
4. Huerre, A., *et al.* Droplets in Microchannels: Dynamical Properties of the Lubrication Film. *Phys. Rev. Lett.* **115**, 064501-064501 (2015).
5. Teletzke, G. F., Davis, H. T. & Scriven, L. E. Wetting hydrodynamics. *Phys. Rev. Appl.* **23**, 989-1007 (1988).
6. Hodges, S. R., Jensen, O. E. & Rallison, J. M. The motion of a viscous drop through a cylindrical tube. *J. Fluid Mech.* **501**, 279-301 (2004).
7. Delgado, A. V., Gonzalezcaballero, F., Hunter, R. J., Koopal, L. K. & Lyklema, J. Measurement and interpretation of electrokinetic phenomena. *J. Colloid Interface Sci.* **309**, 194-224 (2007).
8. Najafi, A. S., Drelich, J., Yeung, A., Xu, Z. & Masliyah, J. A novel method of measuring electrophoretic mobility of gas bubbles. *J. Colloid Interface Sci.* **308**, 344-350 (2007).
9. Kim, J., Song, M. & Kim, J. Zeta Potential of Nanobubbles Generated by Ultrasonication in Aqueous Alkyl Polyglycoside Solutions. *J. Colloid Interface Sci.* **223**, 285-291 (2000).
10. Cho, S. H., Kim, J. Y., Chun, J. H. & Kim, J. D. Ultrasonic formation of nanobubbles and their zeta-potentials in aqueous electrolyte and surfactant solutions. *Colloids Surf., A* **269**, 28-34 (2005).
11. Fan, X., Zhang, Z., Li, G. & Rowson, N. A. Attachment of solid particles to air bubbles in surfactant-free aqueous solutions. *Chem. Eng. Sci.* **59**, 2639-2645 (2004).
12. Behrens, S. H. & Grier, D. G. The charge of glass and silica surfaces. *J. Phys. Chem.* **115**, 6716-6721 (2001).
13. Hanaor, D. A., Ghadiri, M., Chrzanowski, W. & Gan, Y. Scalable surface area characterization by electrokinetic analysis of complex anion adsorption. *Langmuir* **30**, 15143-15152 (2014).
14. Chiou, N., Lu, C., Guan, J., Lee, L. J. & Epstein, A. J. Growth and alignment of polyaniline nanofibres with superhydrophobic, superhydrophilic and other properties. *Nat. Nanotechnol.* **2**, 354-357 (2007).
15. Joly, L., Detcheverry, F. & Biance, A. Anomalous ζ potential in foam films. *Phys. Rev. Lett.* **113**, 088301 (2014).
