# Supplementary material for: Dimension-reconfigurable bubble film nanochannel for wetting based sensing
Source: Nat Commun. 2020 Feb 10;11:814. doi: 10.1038/s41467-020-14580-x (PMC7010761; doi:10.1038/s41467-020-14580-x)
Supplement: Supplementary file 2 — Description of Additional Supplementary Files [file 41467_2020_14580_MOESM2_ESM.pdf]

Description of Additional Supplementary Files

File Name: Supplementary Movie 1
Description: The process of generating micro bubble in PDMS microchannel and glass capillary. We stopped the flows and remained a single microbubble in a capillary by removing the rest of bubbles out of the capillary and microchannels through the branch channels in the PDMS chip.
